# Tobacco Hornworm (*Manduca sexta*) Oral Secretion Elicits Reactive Oxygen Species in Isolated Tomato Protoplasts

**DOI:** 10.3390/ijms21218297

**Published:** 2020-11-05

**Authors:** Akanksha Gandhi, Rupesh R. Kariyat, Cruz Chappa, Mandeep Tayal, Nirakar Sahoo

**Affiliations:** 1 Department of Biology, University of Texas Rio Grande Valley, Edinburg, TX 78539, USA; akanksha.gandhi01@utrgv.edu (A.G.); rupesh.kariyat@utrgv.edu (R.R.K.); cruz.chapa01@utrgv.edu (C.C.); mtayal@g.clemson.edu (M.T.); 2 School of Earth, Environmental and Marine Sciences, University of Texas Rio Grande Valley, Edinburg, TX 78539, USA; 3 Mathematics and Science Academy, University of Texas Rio Grande Valley, Edinburg, TX 78539, USA

**Keywords:** reactive oxygen species, *Manduca sexta*, oral secretions, protoplast, calcium, antioxidant

## Abstract

Plants are under constant attack by a suite of insect herbivores. Over millions of years of coexistence, plants have evolved the ability to sense insect feeding via herbivore-associated elicitors in oral secretions, which can mobilize defense responses. However, herbivore-associated elicitors and the intrinsic downstream modulator of such interactions remain less understood. In this study, we show that tobacco hornworm caterpillar (*Manduca sexta*) oral secretion (OS) induces reactive oxygen species (ROS) in tomato (*Solanum lycopersicum*) protoplasts. By using a dye-based ROS imaging approach, our study shows that application of plant-fed (PF) *M. sexta* OS generates significantly higher ROS while artificial diet-fed (DF) caterpillar OS failed to induce ROS in isolated tomato protoplasts. Elevation in ROS generation was saturated after ~140 s of PF OS application. ROS production was also suppressed in the presence of an antioxidant NAC (*N*-acetyl-*L*-cysteine). Interestingly, PF OS-induced ROS increase was abolished in the presence of a Ca^2+^ chelator, BAPTA-AM (1,2-bis(o-aminophenoxy)ethane-*N*,*N*,*N*′,*N*′-tetraacetic acid). These results indicate a potential signaling cascade involving herbivore-associated elicitors, Ca^2+^, and ROS in plants during insect feeding. In summary, our results demonstrate that plants incorporate a variety of independent signals connected with their herbivores to regulate and mount their defense responses.

## 1. Introduction

Herbivory is an unavoidable part of a plant’s life. Over millions of years, plants and herbivorous insects have been involved in a relentless war where plants are actively attacked by herbivores, reducing plant growth, development, and, consequently, their fitness [1]. It is estimated that insect herbivory leads to about ~20 percent plant growth loss annually [2,3]. To counter this, although sessile, plants have evolved several defense approaches, which include morphological, biochemical, and molecular mechanisms [4,5,6,7,8]. During an insect attack, the host plant perceives at least two types of signals: (1) physical injury or wounding known as damage-associated molecular patterns (DAMPs) and (2) chemical cues found in herbivore oral secretions (OS) or oviposition fluid (OF), known as herbivore-associated molecular patterns (HAMPs) [9,10,11,12,13].

Herbivore-plant interactions are generally initiated at the plant cell membrane, where herbivore-associated elicitors trigger a series of signaling cascades that initiate induced plant responses [14,15,16,17]. It has been proposed that following insect attack the foremost event is plasma membrane potential change (V_m_) [18,19], followed by generation of second messengers such as cytosolic calcium (Ca^2+^) [16,20] and reactive oxygen species (ROS) [21,22,23,24] that facilitate plant defense signal transduction. This leads to a suite of defense-related traits, including induction of trichomes, spines, and secondary metabolites (e.g., alkaloids, phenolics, and volatile organic compounds) that negatively impact herbivore fitness and mediate multi-trophic interactions [7,25,26]. While ecological aspects of plant-herbivore interactions and their molecular mechanisms are well understood, early initiation mechanisms associated with alterations in V_m_, Ca^2+^, and ROS production immediately after herbivore assault warrants more empirical testing in various systems [27].

ROS is a significant biomolecule that plays a crucial role in defense signaling in plants [28,29]. It is well known that there is a rapid generation of molecules such as superoxide (O^−^), hydrogen peroxide (H_2_O_2_), and hydroxyl radicals (HO^−^) upon insect attack that leads to an oxidative burst [30]. Previous studies have shown that plants can identify herbivore OS that leads to oxidative burst and facilitates transmitting long-distance signals [11,13,18,31,32]. ROS production is indispensable for systemic induction of defense responses in plants [28,29]. Regardless of the significance of oxidative signaling in several facets of cell biology, our knowledge of it and its regulation remains limited [33].

In this study, we identified that *Manduca sexta* OS stimulates ROS generation in isolated tomato protoplasts. *M. sexta* (tobacco hornworm) is a crucial insect model used to test both ecological effects and molecular mechanisms underlying plant-herbivore interaction research [7,9,34,35,36]. *M. sexta* is a specialist on Solanaceae, which includes *Solanum lycopersicum* (tomato), which also serves as an excellent cellular model for plant defense-related studies [37]. By utilizing a ROS-sensing dye 2′,7′-dichlorodihydrofluorescein diacetate (CM-H_2_DCFDA)-based cell imaging technique, we efficiently measured transient elevation in ROS generation upon application of *M. sexta* OS. This ROS-sensing dye has been previously used in studying in vivo ROS production in root cells and hairs [38]. Our investigation demonstrates that *M. sexta* OS induces ROS production in tomato protoplasts and the OS effect is altered based on the diet choices of the insect. Moreover, we identified that *M. sexta* OS-mediated ROS generation is dependent on intracellular Ca^2+^.

## 2. Results

### 2.1. M. sexta OS Induces ROS Generation in Tomato Protoplast

**Figure 1:**
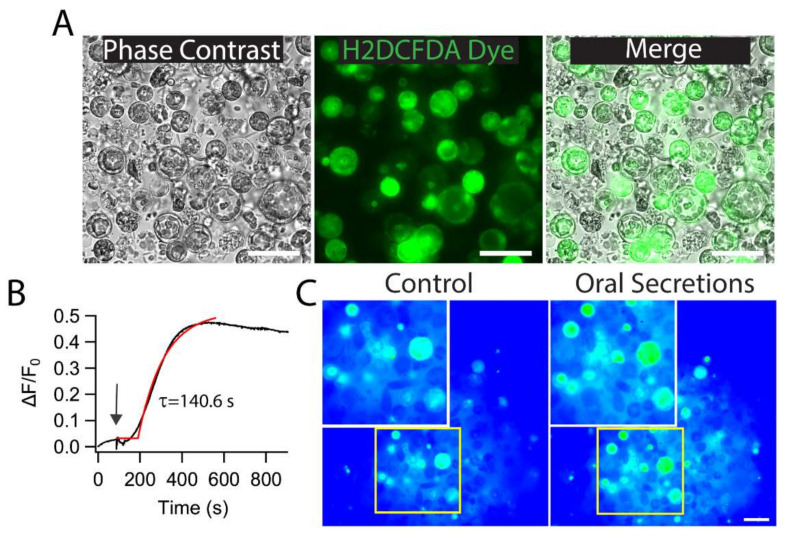
Effect of *M. sexta* oral secretion (OS) on reactive oxygen species (ROS) production in tomato protoplasts. (**A**) Representative phase contrast image (left) of protoplasts at 100 × magnification isolated from tomato leaves. Isolated protoplasts were loaded with ROS-sensing dye CM-H_2_DCFDA (middle). (**B**) Representative ROS imaging trace showing an increase in protoplasts ROS level upon application of *M. sexta* OS. The data were fitted with a single exponential fit function with a lag of 134.2 ± 11.4 s and a τ of 140.5 ± 5.9 s. (**C**) Representative image of ROS generation in tomato protoplast at 40× magnification before and after 400 s of *M. sexta* OS application. Scale bar: 10 µm. The number of protoplasts (*N*) from 3–5 independent measurements is provided in parentheses in (**B**).

While herbivores prey on the plant, protoplasts come into contact with oral secretions that induce plant defense signaling, and ROS has been known to play a critical role in these defense responses. To determine if herbivore OS would modulate ROS levels in the plant, we performed CM-H_2_DCFDA dye-based ROS imaging of tomato protoplast and tested the effect of herbivore *M. sexta* crude OS (Figure 1A). We found that the application of *M. sexta* OS induced a drastic increase of ROS generation in isolated tomato protoplasts. After a lag of 134.2 ± 11.4 s, the ROS level reached a maximum after 140.5 ± 5.9 s of *M. sexta* OS application (Figure 1B,C; *N* = 74). These data indicate that *M. sexta* OS is a potent elicitor of ROS in plant protoplasts.

### 2.2. Diet-Dependent M. sexta OS Effect on ROS Production in Tomato Protoplast

**Figure 2:**
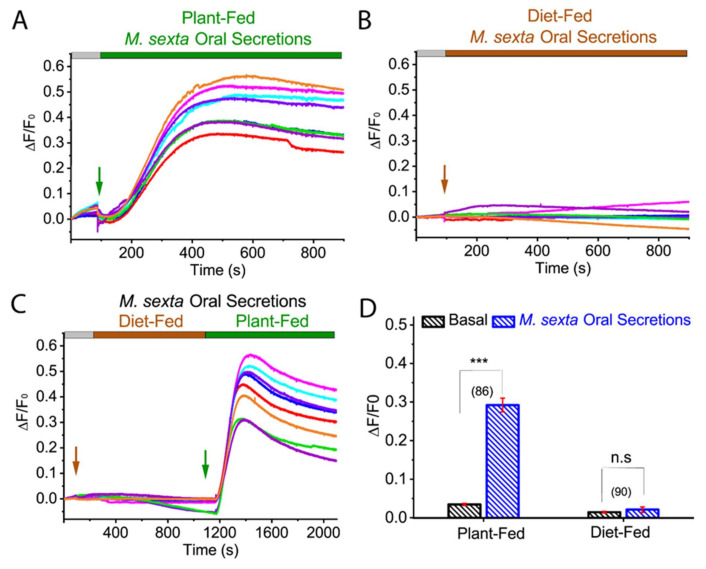
Plant-fed *M. sexta* OS-induced ROS elevation in tomato protoplasts. Representative ROS imaging of tomato protoplasts with the application of tomato plant-fed (PF) *M. sexta* OS (**A**), diet-fed (DF) *M. sexta* OS (**B**), and combination of both (**C**). (**D**) Bar graph analysis of data shown in (**A**,**B**) depicting maximum ROS generation after PF and DF *M. sexta* OS application. Statistical indicators reflect the non-parametric Mann-Whitney test, measuring for an effect of PF and DF *M. sexta* OS on ROS production: n.s, not significant; *** *p* < 0.0001. Different color traces in the graph (**A**–**C**) reflect the OS-induced ROS response in individual protoplasts from a single replicate. The number of protoplasts (*N*) from 3–5 independent measurements are provided in parentheses in (**D**).

Many herbivores have coevolved with specific plant host/s, and typically exhibit preferences to feed on them. On the other hand, these host plants can sense the herbivore-derived elicitor such as OS, regurgitant, and saliva that are composed of host plant materials and use it to facilitate plant defense signal transduction. To investigate whether *M. sexta* OS-mediated ROS increase is diet-dependent, we tested the effect of OS derived from tomato plant-fed (PF) and artificial diet-fed (DF) *M. sexta*. Our ROS imaging recording from the tomato protoplast showed that application of tomato PF *M. sexta* OS increased ROS generation (basal: 0.035 ± 0.003; PF OS: 0.292 ± 0.018; *p* < 0.0001; Mann-Whitney test) (Figure 2A,C,D; *N* = 86) while artificial DF *M. sexta* OS failed to induce ROS in isolated tomato protoplasts (basal: 0.015 ± 0.002; DF OS: 0.021 ± 0.007; *p* = 0.384; Mann-Whitney test) (Figure 2B,C,D; *N* = 90). These results suggest that the herbivore OS diet plays an essential role in the generation of ROS in host plants.

### 2.3. Membrane-Permeable Oxidant tbH_2_O_2_-Induced ROS in Tomato Protoplast

**Figure 3:**
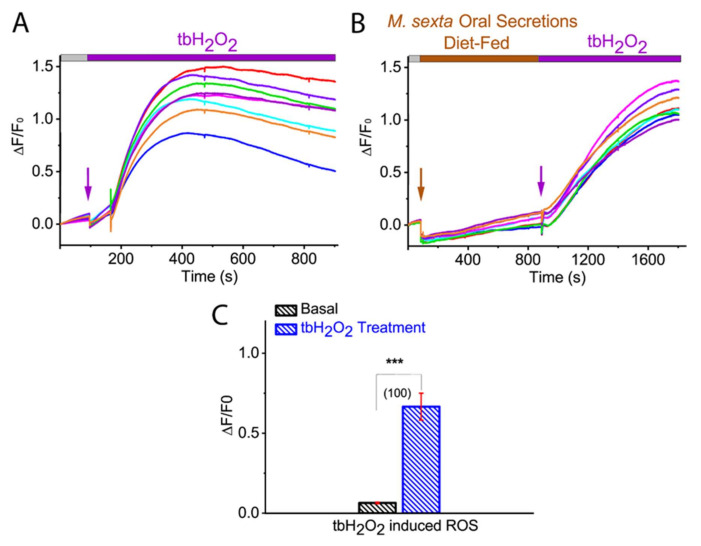
Effect of membrane-permeable oxidant tbH_2_O_2_ on ROS production in tomato protoplasts. Representative ROS imaging of the isolated tomato protoplast with the application of membrane-permeable oxidant tbH_2_O_2_ (**A**) and after application of diet-fed *M. sexta* OS (**B**). (**C**) Bar graph analysis of data shown in (**A**) illustrating maximum ROS generation after tbH_2_O_2_ application. Statistical indicators reflect the non-parametric Mann-Whitney test, measuring for an effect of tbH_2_O_2_ on ROS production: *** *p* < 0.0001. Different color traces in the graph (**A**,**B**) reflect the OS-induced ROS response in individual protoplasts from a single replicate. The number of protoplasts (*N*) from 3–5 independent measurements are provided in parentheses in (**C**).

Earlier studies have shown that an increase in ROS, such as H_2_O_2_ production, was achieved within 5 min of herbivore-induced wounding [16,39]. This observation is in line with our findings, which showed that the maximum ROS generation in tomato protoplast was achieved in less than 3 min of *M. sexta* OS application. To investigate whether our ROS imaging approach could detect H_2_O_2_-induced ROS, we applied a membrane-permeable H_2_O_2_ tert-butyl hydrogen peroxide (tbH_2_O_2_), to the CM-H_2_DCFDA dye loaded tomato protoplasts. As shown in Figure 3, the increase in maximum ROS production was observed after 2 min of application of tbH_2_O_2_ (basal: 0.064 ± 0.005; tbH_2_O_2_: 0.665 ± 0.084; *p* < 0.0001; Mann-Whitney test) (Figure 3A,D; *N* = 100). These results indicate that our ROS imaging approach could efficiently detect intracellular ROS either by H_2_O_2_ or herbivore OS.

### 2.4. Antioxidant N-Acetylcysteine (NAC) Abolished M. sexta OS, and Oxidant tbH_2_O_2_-Induced ROS Generation in Tomato Protoplasts

**Figure 4:**
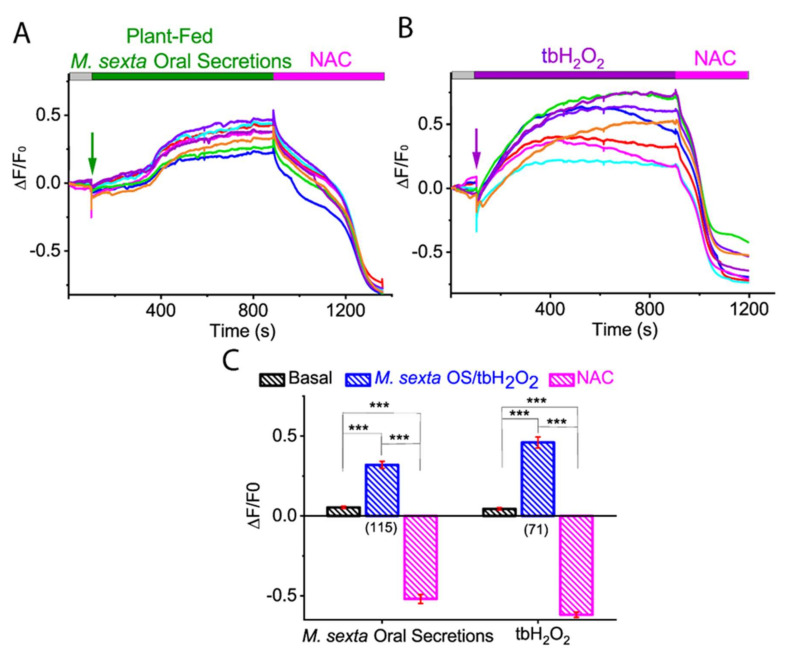
Effect of antioxidant NAC on *M. sexta* OS and oxidant tbH_2_O_2_-induced ROS production in tomato protoplasts. Representative ROS imaging of the isolated tomato protoplast with the application of PF *M. sexta* OS (**A**) and tbH_2_O_2_ (**B**), followed by the application of antioxidant NAC. (**C**) Bar graph analysis of data shown in (**A**,**B**) illustrating the maximum ROS generation after PF *M. sexta* OS and tbH_2_O_2_ application and the minimum ROS level after NAC application. Statistical indicators reflect the non-parametric Kruskal-Wallis test followed by Dunn’s pairwise post hoc comparisons, testing for an effect of PF *M. sexta* OS, tbH_2_O_2_, and NAC on the ROS level in the isolated protoplasts: *** *p* < 0.0001. Different color traces in the graph (**A**,**B**) reflect the OS-induced ROS response in individual protoplasts from a single replicate. The number of protoplasts (*N*) from 3–5 independent measurements are provided in parentheses in (**C**).

The evidence presented so far suggests that *M. sexta* OS and tbH_2_O_2_ induce ROS generation in the isolated tomato protoplast. To further validate these observations, we applied a membrane-permeable antioxidant *N*-acetyl-cysteine (NAC), a glutathione (GSH) precursor that boosts GSH content in cells. As shown in Figure 4, application of NAC to the tomato protoplast efficiently quenched ROS generated by *M. sexta* OS (basal: 0.048 ± 0.006; PF OS: 0.319 ± 0.019; NAC: −0.552 ± 0.026; *p* < 0.0001; Kruskal-Wallis test, followed by Dunn’s pairwise posthoc analysis) (Figure 4A,C; *N* = 115) and tbH_2_O_2_ (basal: 0.043 ± 0.006; tbH_2_O_2_: 0.460 ± 0.034; NAC: −0.619 ± 0.016; *p* < 0.0001; Kruskal-Wallis test followed by Dunn’s pairwise posthoc analysis) (Figure 4B,C; *N* = 71). However, NAC treatment led to a negative baseline, suggesting that protoplasts were partially oxidized in our experimental conditions (Figure 4 and Supplementary Figure S1). This finding further supports that *M. sexta* OS is a ROS inducer in isolated protoplasts.

### 2.5. Calcium Chelator BAPTA-AM Inhibited M. sexta OS-Induced ROS Generation in Tomato Protoplasts

**Figure 5:**
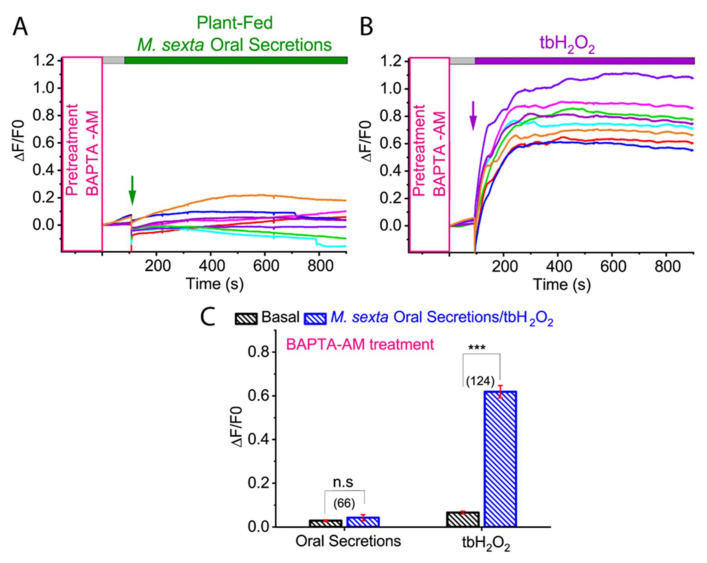
Effect of Ca^2+^ chelator BAPTA-AM on *M. sexta* OS and tbH_2_O_2_-induced ROS generation in tomato protoplasts. Representative ROS imaging of the isolated tomato protoplast in the presence of BAPTA, with the application of PF *M. sexta* OS (**A**) and tbH_2_O_2_ (**B**). (**C**) Bar graph analysis of data shown in (**A**,**B**) illustrating the maximum ROS generation after PF *M. sexta* OS and tbH_2_O_2_ application. Statistical indicators reflect the non-parametric Mann-Whitney test, measuring for an effect of PF *M. sexta* OS and tbH_2_O_2_ on ROS level in the BAPTA-AM preincubated isolated protoplasts: n.s, not significant; *** *p* < 0.0001. Different color traces in the graph (**A**,**B**) reflect the OS-induced ROS response in individual protoplasts from a single replicate. The number of protoplasts (*N*) from 3–5 independent measurements are provided in parentheses in (**C**).

Calcium (Ca^2+^) has been known to serve as a second messenger in plant-herbivore interactions. Several studies have shown that herbivore-induced wounding triggers a dramatic Ca^2+^ cytosolic ion influx, which further regulates the formation of ROS [16]. To investigate whether *M. sexta* OS-induced ROS generation is dependent on cytosolic Ca^2+^, we pre-incubated tomato protoplasts in BAPTA-AM, a membrane-permeable Ca^2+^ chelator and tested the effect of *M. sexta* OS on ROS generation. As shown in Figure 5A,C, application of *M. sexta* OS in BAPTA-AM pre-incubated tomato protoplasts inhibited ROS production (basal: 0.028 ± 0.003; PF OS: 0.042 ± 0.013; *p* = 0.786; Mann-Whitney test) (Figure 5A,C; *N* = 66). However, tbH_2_O_2_-induced ROS was not affected by Ca^2+^ chelator BAPTA-AM (basal: 0.066 ± 0.006; tbH_2_O_2_: 0.618 ± 0.028; *p* < 0.001; Mann-Whitney test) (Figure 5B,C; *N* = 124). These results indicate that *M. sexta* OS-induced ROS generation was mediated by cytosolic Ca^2+^.

## 3. Discussion

Identification of herbivore elicitors and their regulation of intracellular ROS production is vital for unraveling non-self-recognition signaling cascades in plants. In this study, we show that OS from *M. sexta* is effective in producing ROS in tomato protoplasts and OS-induced intracellular ROS production is dependent on intracellular Ca^2+^. Our results of ROS imaging of a single protoplast to understand the kinetics of ROS initiation upon herbivore OS application will be critical in understanding early initiation events in herbivore defenses in plants. Our cellular approach of dissecting ROS plays an essential part in various pathways, including physiological, hormonal, and developmental aspects of plant growth [12,13]. In addition, ROS also plays a crucial role in the defense signaling cascade against abiotic and biotic stress conditions [40,41,42,43,44]. Hence, understanding ROS in plants remains an emerging field of research. More recently, several studies have used fluorescent reporter molecules to measure ROS levels in vivo and have collectively documented that these molecules are robust and promising tools that can measure ROS in real-time with high sensitivity [45,46,47,48]. However, these probes, including diaminobenzidine (DAB), nitro blue tetrazolium (NBT), and Amplex Red, have certain limitations of being toxic and susceptible to degradation by light [49]. However, use of CM-H_2_DCFDA in protoplasts is a valuable ROS indicator to study plant-herbivore interactions [38,48]. A study by Maffei et al. showed that ROS (H_2_O_2_) accumulation was observed in lima bean leaves (*Phaseolus lunatus*) incubated with DAB upon attack by *Spodoptera littoralis* and in mechanically damaged leaves [45]. However, H_2_O_2_ production occurred more in herbivore-wounded zones in comparison to mechanically damaged leaves. To further validate the finding, CM-H_2_DCFDA-dye with confocal laser scanning microscopy was used, which confirmed variation in H_2_O_2_ generation in mechanically damaged and herbivore-wounded leaves. In addition, a recent study by Fischman et al. showed local and systemic ROS signal accumulation upon wounding and was evaluated by using CM-H_2_DCFDA dye-based ROS sensing in whole plants [50]. This new method of examining ROS generation on whole mature plants in real-time could unravel systemic signaling in plants and greatly facilitate the identification of new pathways for ROS signaling. Our study clearly demonstrates that a CM-H_2_DCFDA dye-based ROS imaging approach on a single tomato protoplast was able to quantify and visualize ROS generation without any toxic effects on cell health.

Among the signaling molecules leading to defense induction, ROS was found to be crucial, and the timing of ROS generation plays a vital role in initiating plant responses. For example, ROS (H_2_O_2_) was generated less than 5 min after herbivore-induced damage [16,39,51,52]. In another study by Mohanta et al., the generation of ROS (H_2_O_2_) in the maidenhair tree (*Ginkgo biloba*) was observed after 30 min upon herbivory by Egyptian cotton leafworm (*Spodoptera littoralis*) [53]. These observations are in line with our findings: our cellular approach found that the maximum ROS generation in the tomato protoplast was achieved in less than 3 min of *M. sexta* OS application. Clearly, regardless of feeding habit (chewing or sucking mouthparts), ROS is critical. This is in addition to the upregulation of genes associated with oxidative stress, along with Ca^2+^ signaling [54,55,56]. Previous studies have reported that plants perceive components that are mainly of plant origin once they are encountered by the herbivore [57]. Our results have added a new dimension to the previously known fact that OS from plant origin (PF OS) can induce ROS signals while OS from artificial diet (DF OS) do not generate ROS, thus giving an indication that PF OS contains components that are responsible for stimulating ROS in protoplasts. It is possible that differences in ROS responses to OS from plant feeding and diet feeding could be due to plant components such as fragments of the cell wall. It will be interesting to tease out various OS components and understand which of them are mainly responsible for ROS generation. Clearly, herbivore diet plays a crucial role in plant defense signaling, an area we are currently exploring in detail using mass spectrometry to examine differences in the composition of both PF and DF OS from *M. sexta* and other herbivore species feeding on different plant species.

Elicitor-dependent production of secondary messengers such as ROS and Ca^2+^ is critical to several signaling processes in plants [17,58,59,60]. Nevertheless, details of mechanisms that control mutual interrelation of ROS and Ca^2+^ signaling are merely starting to emerge. One of the fascinating questions is whether ROS production is interconnected to Ca^2+^ signaling [61]. In order to unravel this, we used BAPTA-AM, which is the most commonly used Ca^2+^ chelator in mammalian cells. Application of PF OS in the presence of BAPTA-AM on the isolated tomato protoplasts failed to show ROS accumulation, indicating the mechanistic link between Ca^2+^ and ROS production. Studies have shown that ROS is regulated by intracellular Ca^2+^ [62,63,64]. Upon insect attack, a first “priming” Ca^2+^ inflow occurs, followed by the release of Ca^2+^ from intracellular stores such as vacuole and mitochondria via Ca^2+^ channels. An increase in cytoplasmic Ca^2+^ activates NADPH oxidases, an enzyme responsible for ROS generation upon binding of Ca^2+^ to its EF hands motifs, resulting in plant defense responses [65,66,67]. ROS could also activate Ca^2+^ channels and facilitate ROS-mediated Ca^2+^ fluxes [62,63]. These ROS-dependent events could initiate a cellular amplification loop, resulting in Ca^2+^ wave propagation from cell-to-cell communication. Our results support the possible connection between the ROS-Ca^2+^ signaling pathway that might be helpful in understanding plant-herbivore interactions at the cellular level.

We chose tbH_2_O_2_ over H_2_O_2_ to study the ROS response in isolated tomato protoplasts because H_2_O_2_ gets quickly oxidized and produces small bubbles in solutions containing a protoplast, which rendered difficulties in measuring ROS responses in our experimental condition. In addition, H_2_O_2_ is very slowly permeable across the membrane. Therefore, we used a membrane-permeable version, tbH_2_O_2_, which showed a strong ROS response to CM-H_2_DCFDA loaded tomato protoplasts. To investigate further the ROS response mediated via *M. sexta* OS and tbH_2_O_2_, we used a membrane-permeable antioxidant, NAC, that has a free radical scavenging property, and which is frequently used in animal ROS research. It resulted in suppression of *M. sexta* OS and tbH_2_O_2_-induced ROS production. Our study has depicted, for the first time, the use of these two chemicals: (1) membrane-permeable oxidant tbH_2_O_2_ and (2) membrane-permeable antioxidant NAC in a plant system and could be used in plant-herbivore interaction research.

## 4. Materials and Methods

### 4.1. Plant Material

F1 tomato hybrid seeds (variety: Valley Girl, Johnny’s Selected Seeds, Fairfield, ME, USA) were grown in pots in a growth chamber at 25 °C with a relative humidity of 65%. The seeds were sown in Sunshine professional growing mix (Sun Gro Horticulture Canada Ltd., Agawam, MA, USA). Seedlings were transplanted two weeks after germination, and OMRI (Organic Material Review Institute, Eugene, OR, USA) listed organic fish emulsion fertilizer (NPK 5:1:1, Alaska Fish Fertilizer, Pennington Seed, Inc., Madison, GA, USA) was added once in two weeks. Plants were watered regularly and grown in controlled conditions without herbivores [35]. All plants used in the study were 4 weeks old after transplanting.

### 4.2. Protoplast Isolation

Protoplasts were isolated by modifying the method described by Zhai et al. [68]. Briefly, 0.5 g of the leaf material from 4-week-old tomato plant was collected and sliced using a fresh razor blade in 3.75 mL of the TVL solution (0.3 M sorbitol and 50 mM CaCl_2_). This solution was stored at −20 °C until further use. Following this, 5 mL of the enzyme solution containing 0.5 M sucrose, 10 mM MES-KOH [pH 5.7], 20 mM CaCl_2_, 40 mM KCl, 0.9% macerozyme and 1.5% cellulase (Research Products International Corp., Mt. Prospect, IL, USA) was prepared and heated at 55 °C to inactivate proteases and increase enzyme solubility. Finely chopped leaf tissue was transferred to a beaker with enzyme solution that was freshly prepared to retain the efficiency of the enzymes. The beaker was covered with aluminum foil and parafilm and was subjected to vacuum for 15 min [69]. The plant tissues were then kept on a shaker at 35 rpm in the dark for 12–14 h. After overnight shaking, the digested material was filtered through eight-layered cheesecloth, pre-wet in W5 solution (0.1% (*w*/*v*) glucose, 0.08% (*w*/*v*) KCl, 0.9% (*w*/*v*) NaCl, 1.84% (*w*/*v*) CaCl_2_, 2 mM MES-KOH pH 5.7). The cheesecloth was washed again with 3.75 mL of W5 solution to sieve the remaining protoplasts. The protoplasts were centrifuged for 7 min at 100× *g*. The supernatant was discarded, and the collected pellet was dissolved in 500 µL of W5 solution.

### 4.3. Manduca sexta Rearing and Oral Secretion Collection

Eggs of *M. sexta* (Lepidoptera: Sphingidae) were obtained from a commercial vendor (Great Lake Hornworm Ltd., Romeo, MI, USA) and were hatched in a petri dish containing moist filter paper in a growth chamber (16:8 h light: dark; 25: 22 °C day: night; 65% RH). In order to collect DF and PF OS, half of the first instar larvae were reared on wheat germ-based artificial diet (wheat germ, casein, sucrose, cholesterol, salts, vitamins, agar, and preservatives) purchased from Carolina Biological, Burlington, NC, USA, while the other half were reared on tomato plants [8]. Regurgitant was collected from the oral cavity of newly molted fourth instar larvae by holding the *M. sexta* and gently squeezing its head into a capillary tube/and or an Eppendorf tube was placed at the mouth of *M. sexta*. The collected OS was centrifuged, and the supernatant was stored at −80 °C until further use. For more details of *M. sexta* care, see [35] and OS see [70].

### 4.4. ROS Measurements

ROS measurements were performed at room temperature with the PTI EasyRatioPro system v3.4 (HORIBA Scientific, Piscataway, NJ, USA). Isolated protoplasts were incubated with 2 µM ROS-sensing dye, CM-H_2_DCFDA (2′,7′-dichlorodihydrofluorescein diacetate) (Invitrogen™ Molecular Probes™, Eugene, OR, USA) for 1 h in the dark. A small drop of the protoplast sample carrying ~30–50 protoplasts was placed on a glass coverslip under an Olympus IX71 inverted microscope attached with PTI EasyRatioPro system. A change in fluorescence of a single protoplast was recorded with EasyRatioPro v3.4 software with an excitation wavelength at 494 nm and an emission wavelength at 520 nm. For all chemicals, such as *M. sexta* OS (crude), tbH_2_O_2_ (1 M), and NAC (1 mM), 1–4 uL were dropped into the protoplast sample during live measurements to test their effect on intracellular ROS generation. For the Ca^2+^-dependent ROS generation experiment, isolated protoplasts were preincubated with 1.5 µM of BAPTA-AM (1,2-bis(o-aminophenoxy) ethane-*N*,*N*,*N′*,*N′*-tetraacetic acid) (Invitrogen™ Molecular Probes™) for 1 h prior to ROS measurement.

### 4.5. Data Analysis and Presentation

ROS imaging data were analyzed with EasyRatioPro (PTI, HORIBA Scientific) software and further processed with Excel (Microsoft, Redmond, WA, USA) and Igor Pro v8.0 (Wavemetrics, Lake Oswego, OR, USA) software. Protoplast images were processed with ImageJ (NIH). Figures were prepared with Origin Pro v2020 (Originlab, Northampton, MA, USA) and Adobe Illustrator v24.1 (Adobe, San Jose, CA, USA). Averaged data are presented as means ± SEM (*N* = number of protoplasts from 3–5 independent measurements). For comparisons with two groups such as basal ROS levels and ROS levels from DF *M. sexta* OS and tomato PF *M. sexta* OS, we used the non-parametric Mann-Whitney U test. For comparison with three groups, as depicted in Figure 4, for basal OS/tbH_2_O_2_ and NAC, we used a non-parametric Kruskal-Wallis test followed by Dunn’s pairwise post hoc comparisons. Non-parametric tests were used since data failed to meet normality assumptions after transformations. For all analyses, data from extractions were pooled to attain a sample size of 66–124 protoplasts and were repeated for at least three replications. All analyses were carried out using GraphPad Prism v9.0 (La Jolla, CA, USA).

## 5. Conclusions

Our study identified that *M. sexta* OS is a ROS elicitor and possibly regulates defenses against insect herbivores. Remarkably, the OS effect was dependent on the larval diet of *M. sexta,* while PF OS-induced ROS and DF OS failed to generate ROS, indicating a potential evolutionary divergence of induced resistance in plants. We speculate that variation of primary and secondary species-specific metabolites plays a major role in OS composition, and it is also plausible to expect that OS components of generalist vs. specialist and chewing vs. sucking mouthparts could also covary with their host plants. This study also reported two chemicals, (1) membrane-permeable ROS tbH_2_O_2_ and (2) antioxidant NAC, which could be efficiently employed in dissecting the role of intracellular ROS in plant-herbivore interaction research, a novel cell biology approach in plant-herbivore studies. Furthermore, our study identified that *M. sexta* OS-induced ROS production was Ca^2+^-dependent, suggesting crosstalk between the Ca^2+^ and ROS signaling pathway. Collectively, these data indicate that a herbivore-associated elicitor increased ROS production, which could be a key starting player in the plant defense line up.

## Supplementary Materials

The following are available online at https://www.mdpi.com/1422-0067/21/21/8297/s1, Figure S1: Basal level ROS was quenched by antioxidant “NAC”.
