# Supplementary material for: Tobacco Hornworm (Manduca sexta) Oral Secretion Elicits Reactive Oxygen Species in Isolated Tomato Protoplasts
Source: Int J Mol Sci. 2020 Nov 5;21(21):8297. doi: 10.3390/ijms21218297 (PMC7663960; doi:10.3390/ijms21218297)
Supplement: Supplementary file 1 [file ijms-21-08297-s001.pdf]

International Journal of
Molecular Sciences

MDPI

Supplementary Figure

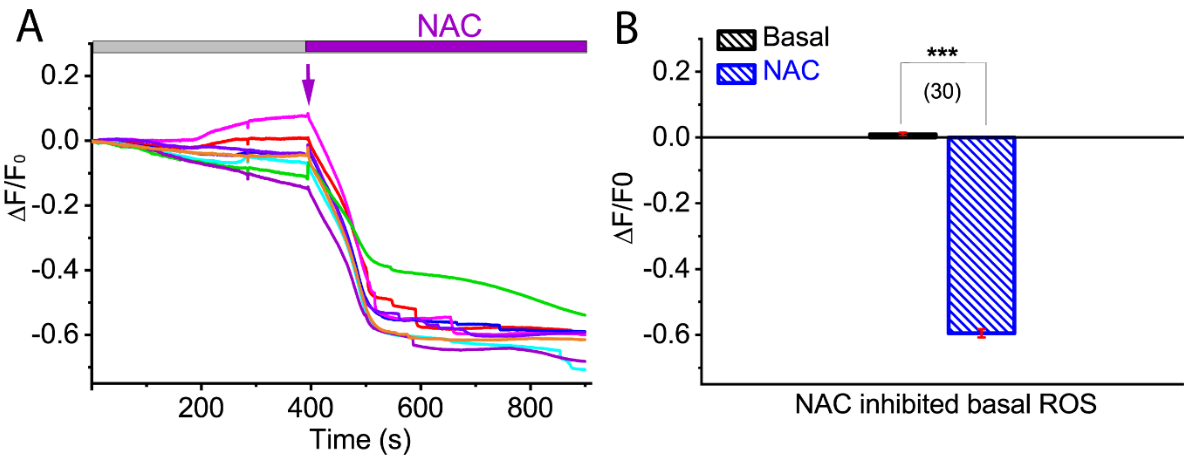

Supplementary Figure S1: Effect of NAC on basal ROS level in tomato protoplasts. **(A)** Representative ROS imaging of isolated tomato protoplast with the application of the antioxidant "NAC" **(B)** Bar graph analysis of data shown in (**A**) illustrating the minimum ROS level after NAC application. Statistical indicators reflect the non-parametric Mann-Whitney test, measuring for an effect of NAC on ROS level: ***$p < 0.0001$.
